# Supplementary material for: Assessing the national essential medicines list selection processes: Instrument development and testing
Source: PLoS One. 2026 Feb 25;21(2):e0342750. doi: 10.1371/journal.pone.0342750 (PMC12935209; doi:10.1371/journal.pone.0342750)
Supplement: S1 File — (DOCX) [file pone.0342750.s001.docx]

**Supplementary Information**

**eTable 1.** Pilot study sample countries

| **Test Countries** | **World Bank income regions** | **World Bank Regions** | **WHO regions** |
| --- | --- | --- | --- |
| **Afghanistan** | Low-income | South Asia | South-east Asia Region |
| **Papua New Guinea** | Low-middle income | East Asia & Pacific | Western Pacific Region |
| **South Africa** | Upper-middle income | Sub-Saharan Africa | African Region |
| **Barbados** | High income | Latin America & Caribbean | Region of the Americas |

**eTable 2:** Item Conversion Factor and score conversion calculation

| **Instrument Item #** | **Original Max Rating** | **Conversion Factor** | **New Max Rating** |
| --- | --- | --- | --- |
| 1 | 5 | 1.0 | 5.0 |
| 2 | 3 | 1.7 | 5.0 |
| 3 | 2 | 2.5 | 5.0 |
| 4 | 2 | 2.5 | 5.0 |
| 5 | 4 | 1.3 | 5.0 |
| 6 | 1 | 5.0 | 5.0 |
| 7 | 1 | 5.0 | 5.0 |
| 8 | 1 | 5.0 | 5.0 |
| 9 | 1 | 5.0 | 5.0 |
| 10 | 4 | 1.3 | 5.0 |
| 11 | 2 | 2.5 | 5.0 |
| 12 | 3 | 1.7 | 5.0 |
| 13 | 4 | 1.3 | 5.0 |
| 14 | 5 | 1.0 | 5.0 |
| 15 | 2 | 2.5 | 5.0 |
| 16 | 2 | 2.5 | 5.0 |
| **Total Score** | **42** |  | **80.0** |

Definitions:

Original Max Rating = the Maximum score for the individual item based on the rating scheme

Conversion Factor = converting the Original Maximum rating for that item to the New Max Rating (5).

New Max Rating = All items should have a New Max Rating of 5

Item Conversion Factor:

$Conversion Factor= \frac{New Max Rating}{Original Max Rating}$

Example of Item score conversion using the conversion factor:

Item 2:

Original Max Rating = 3

New Max Rating = 5

Conversion Factor = 5/3 = 1.7

Therefore, all raw scores for item 2 will be multiplied by the conversion factor.

Raw Item 2 score = 2/3

New Item 2 score = (2) x (5/3) = 3.3

**eFigure 1.** Iterative Item reduction, and revision

25 Evaluation Criteria

25 Evaluation Criteria

Evaluation criteria reworded to be generalized enough for a variety of processes.

17 Evaluation Criteria

8 Evaluation criteria removed due to redundancy.

17 Evaluation Criteria

Evaluation criteria matched to appropriate WHO recommendation

16 Evaluation Criteria

1 Evaluation criteria removed as its focus was on implementation rather than intent.

Notes:

Evaluation criteria reworded to be generalized enough for a variety of processes.

Original: Evidence of clear communication of decision to the public/healthcare personnel/member states

Instrument item: The documentation associated with the decision-making process, such as meeting minutes, is made publicly available.

Evaluation criteria removed due to redundancy: removed items when similar items assessed the identical evaluation criteria

Item Removed: Evidence of selection of best alternative.

Instrument Item kept: Explicit and detailed selection criteria for essential medicines list selection exist.

Evaluation Criteria matched to appropriate WHO recommendation:

WHO Recommendation wording: A review of the clinical guidelines and the list should be carried out at least every second year.

Instrument Evaluation Criteria: Selected EML Revised/Reviewed regularly (There are instructions to review/revise the NEML regularly).

Evaluation criteria removed as its focus was on implementation rather than intent: To maintain focus on policy design any item that focused on process implementation was removed.

**eTable 3:** Raw country scores by item for each rater (R1 to R5)

| ITEM 1: Explicit instructions for the selection of an expert committee exist. | | | | | |
| --- | --- | --- | --- | --- | --- |
| Country | R1 | R2 | R3 | R4 | R5 |
| Afghanistan | 2 | 1 | 1 | 1 | 1 |
| Barbados | 5 | 4 | 4 | 4 | 4 |
| PNG | 0 | 0 | 0 | 0 | 0 |
| South Africa | 5 | 4 | 5 | 4 | 5 |
| ITEM 2: The names, affiliations, and conflict of interest statements of expert committee members are publicly available. | | | | | |
| Country | R1 | R2 | R3 | R4 | R5 |
| Afghanistan | 2 | 2 | 2 | 2 | 2 |
| Barbados | 2 | 1 | 2 | 2 | 1 |
| PNG | 0 | 0 | 0 | 0 | 0 |
| South Africa | 3 | 1 | 3 | 1 | 1 |
| ITEM 3: The expert committee responsible for National Medicines List (NEML) selection operates with full scientific independence. | | | | | |
| Country | R1 | R2 | R3 | R4 | R5 |
| Afghanistan | 0 | 0 | 1 | 1 | 0 |
| Barbados | 0 | 1 | 0 | 1 | 0 |
| PNG | 0 | 0 | 0 | 0 | 0 |
| South Africa | 1 | 1 | 2 | 1 | 1 |
| ITEM 4: Detailed guidelines/principles for the expert committee to establish an essential medicines list exist. | | | | | |
| Country | R1 | R2 | R3 | R4 | R5 |
| Afghanistan | 2 | 2 | 2 | 2 | 2 |
| Barbados | 0 | 2 | 2 | 1 | 2 |
| PNG | 0 | 0 | 1 | 0 | 1 |
| South Africa | 2 | 2 | 2 | 2 | 1 |
| ITEM 5: Explicit and detailed selection criteria for essential medicines list selection exist. | | | | | |
| Country | R1 | R2 | R3 | R4 | R5 |
| Afghanistan | 4 | 4 | 2 | 3 | 3 |
| Barbados | 2 | 2 | 2 | 3 | 3 |
| PNG | 2 | 1 | 1 | 1 | 0 |
| South Africa | 4 | 4 | 4 | 4 | 4 |
| ITEM 6: There is explicit direction to base EM selection decisions on scientific evidence of efficacy and safety, as per the selection criteria. | | | | | |
| Country | R1 | R2 | R3 | R4 | R5 |
| Afghanistan | 1 | 1 | 1 | 1 | 1 |
| Barbados | 0 | 1 | 1 | 1 | 1 |
| PNG | 1 | 1 | 1 | 1 | 1 |
| South Africa | 1 | 1 | 1 | 1 | 1 |
| ITEM 7: The prevalence of health conditions and resistance patterns are considered in EML selection, as per the selection criteria. | | | | | |
| Country | R1 | R2 | R3 | R4 | R5 |
| Afghanistan | 1 | 1 | 1 | 0 | 1 |
| Barbados | 0.5 | 1 | 1 | 1 | 1 |
| PNG | 1 | 1 | 0 | 1 | 1 |
| South Africa | 1 | 1 | 1 | 1 | 1 |
| ITEM 8: The selection criteria of EMs explicitly considers financial implications when examining medicines with equal safety and efficacy. | | | | | |
| Country | R1 | R2 | R3 | R4 | R5 |
| Afghanistan | 1 | 1 | 1 | 0 | 1 |
| Barbados | 1 | 1 | 1 | 1 | 1 |
| PNG | 0 | 1 | 0 | 1 | 1 |
| South Africa | 1 | 1 | 1 | 1 | 1 |
| ITEM 9: The selection criteria of essential medicines assesses the feasibility of uptake (health care setting, personnel etc.). | | | | | |
| Country | R1 | R2 | R3 | R4 | R5 |
| Afghanistan | 0 | 1 | 0 | 0 | 1 |
| Barbados | 1 | 0 | 0 | 1 | 1 |
| PNG | 1 | 1 | 0 | 1 | 1 |
| South Africa | 0 | 1 | 1 | 1 | 1 |
| ITEM 10: There is clear evidence of a National Medicines Policy (NMP) explicitly emphasizing a focus on communication of NEML and clinical guidelines to the public and healthcare personnel. | | | | | |
| Country | R1 | R2 | R3 | R4 | R5 |
| Afghanistan | 3 | 2 | 3 | 3 | 2 |
| Barbados | 4 | 4 | 4 | 4 | 3 |
| PNG | 3 | 3 | 2 | 3 | 1 |
| South Africa | 4 | 4 | 2 | 4 | 3 |
| ITEM 11: The documentation associated with the decision-making process, such as meeting minutes, is made publicly available. | | | | | |
| Country | R1 | R2 | R3 | R4 | R5 |
| Afghanistan | 1 | 0 | 2 | 1 | 1 |
| Barbados | 1 | 0 | 0 | 1 | 0 |
| PNG | 0 | 0 | 0 | 0 | 0 |
| South Africa | 1 | 2 | 2 | 2 | 2 |
| ITEM 12: The selection process used to select EMs is published publicly. (Website, journal, industry paper, etc.) | | | | | |
| Country | R1 | R2 | R3 | R4 | R5 |
| Afghanistan | 3 | 3 | 2 | 1 | 2 |
| Barbados | 0 | 3 | 2 | 1 | 2 |
| PNG | 0 | 0 | 1 | 0 | 0 |
| South Africa | 3 | 3 | 3 | 3 | 3 |
| ITEM 13: There is a means for the public or other interested parties to question decisions on inclusion/exclusion of Essential Medicine on the NEML. | | | | | |
| Country | R1 | R2 | R3 | R4 | R5 |
|  |  |  |  |  |  |
| Afghanistan | 3 | 2 | 2 | 3 | 2 |
| Barbados | 2 | 1 | 3 | 1 | 2 |
| PNG | 1 | 0 | 1 | 0 | 1 |
| South Africa | 4 | 3 | 2 | 3 | 3 |
| ITEM 14: There are clear indications that the EM selection process is reviewed (external/internal review of information). | | | | | |
| Country | R1 | R2 | R3 | R4 | R5 |
| Afghanistan | 3 | 1 | 1 | 3 | 0 |
| Barbados | 0 | 0 | 0 | 2 | 0 |
| PNG | 0 | 4 | 1 | 4 | 4 |
| South Africa | 4 | 1 | 5 | 1 | 5 |
| ITEM 15: Selected EML Revised/Reviewed regularly (There are instructions to review/revise the NEML regularly). | | | | | |
| Country | R1 | R2 | R3 | R4 | R5 |
| Afghanistan | 1 | 1 | 2 | 1 | 2 |
| Barbados | 2 | 2 | 2 | 2 | 0 |
| PNG | 2 | 0 | 1 | 0 | 0 |
| South Africa | 2 | 2 | 2 | 1 | 2 |
| ITEM 16: The use and impact of EML implementation is monitored. (There are instructions to monitor the use and impact of the NEML as a policy tool.) | | | | | |
| Country | R1 | R2 | R3 | R4 | R5 |
| Afghanistan | 0 | 0 | 2 | 1 | 1 |
| Barbados | 2 | 2 | 2 | 1 | 0 |
| PNG | 2 | 2 | 2 | 2 | 0 |
| South Africa | 2 | 1 | 2 | 2 | 2 |

**R1-R5 – 5 raters that rated all four pilot countries using the 16-item instrument.**

**eTable 4:** Total country scores

Total Raw Score for each pilot study country by each rater.

| TOTAL – RAW SCORE | | | | | |
| --- | --- | --- | --- | --- | --- |
| Country | R1 | R2 | R3 | R4 | R5 |
| Afghanistan | 27 | 22 | 25 | 23 | 22 |
| Barbados | 22.5 | 25 | 26 | 27 | 21 |
| PNG | 13 | 14 | 11 | 14 | 11 |
| South Africa | 38 | 32 | 38 | 32 | 36 |

Total percentage score after conversion for pilot study country by each rater.

| TOTAL – PERCENTAGE SCORE | | | | | |
| --- | --- | --- | --- | --- | --- |
| Country | R1 | R2 | R3 | R4 | R5 |
| Afghanistan | 64% | 52% | 60% | 55% | 52% |
| Barbados | 54% | 60% | 62% | 64% | 50% |
| PNG | 31% | 33% | 26% | 33% | 26% |
| South Africa | 90% | 76% | 90% | 76% | 86% |

**eTable 5**: Validity testing raw and percentage data

| **Measure 1** | | | |
| --- | --- | --- | --- |
| Country | # medicines added WHO model EML | common medicines on corresponding NEML | common medicines on corresponding NEML(%) |
| Afghanistan 2014 | 17 | 7 | 53% |
| Barbados 2012 | 16 | 9 | 56% |
| Papua New Guinea 2012 | 16 | 1 | 6% |
| South Africa 2014 | 17 | 9 | 53% |
| **Measure 2** | | | |
|  | #medicines deleted WHO model EML | medicine still on corresponding NEML | medicine still on corresponding NEML (%) |
| Afghanistan 2014 | 2 | 1 | 50% |
| Barbados 2012 | 15 | 4 | 27% |
| Papua New Guinea 2012 | 15 | 5 | 33% |
| South Africa 2014 | 2 | 1 | 50% |
| **Validity Test Compiled Data for all 3 measures** | | | |
|  | NEML design effectiveness score | Added meds common between WHO model EML and NEML (%) | Deleted meds from WHO model EML still on NEML (%) |
| Afghanistan 2014 | 57% | 53% | 50% |
| Barbados 2012 | 58% | 56% | 27% |
| Papua New Guinea 2012 | 30% | 6% | 33% |
| South Africa 2014 | 83% | 53% | 50% |
