# Supplementary material for: Assessing the national essential medicines list selection processes: Instrument development and testing
Source: PLoS One. 2026 Feb 25;21(2):e0342750. doi: 10.1371/journal.pone.0342750 (PMC12935209; doi:10.1371/journal.pone.0342750)
Supplement: Attachment — Submitted filename: NEML Review.docx [file pone.0342750.s003.docx]

I have reviewed the manuscript, but major comments need to be addressed accordingly, the manuscript addresses an important gap by proposing and validating an instrument to assess the design effectiveness of NEML selection processes. However, some methodological, conceptual, and reporting issues require attention before publication:

1. Title of study should be revised for better clarity, it should be catchy and concise
2. Background is long, make it short max 2 lines
3. Purpose/aim/objective of the study should be mentioned separately after the background.
4. The abstract reports ICC values but does not specify the pilot sample size (n=4 countries, 5 raters). Including this would improve transparency.
5. Key words should be mentioned alphabetically
6. Abbreviations should be written in full form first e.g. AFR, EML, NEML, NPPs etc, check this throughout the manuscript.
7. Ethics approval waiver letter is required, mention the details of waiver in revised manuscript and also provide the waiver letter to reviewers and editorial office for their perusal and consideration.
8. While the study used publicly available documents, the authors should clarify whether consultation with WHO experts and recruited raters required institutional ethics approval or exemption (particularly since human raters were involved). Currently, this section is minimal.
9. The authors claim this is the first validated instrument to assess NEML selection process design. While the novelty is clear, the manuscript could benefit from a deeper comparison with existing frameworks/tools for priority setting and policy evaluation e.g., WHO checklist approaches, health policy analysis frameworks. This would better situate the contribution.
10. Why Only four countries (Afghanistan, Papua New Guinea, South Africa, Barbados) were assessed? The rationale for their selection is only briefly explained (income-level stratification). A stronger justification is needed for why these specific countries were chosen and how representative they are. Small sample size also raises concerns about generalizability.
11. While total instrument reliability is excellent (ICC > 0.9), sub-domain reliability (e.g., Relevance, Revisions & Appeals, Enforcement) is weak due to wide CIs and limited items. The authors should discuss how this affects interpretability of subscale scores and whether additional items are needed to improve measurement stability.
12. It was observed that the proxy measures “alignment with WHO model EML additions/removals are reasonable but limited. They primarily capture timeliness rather than overall process quality. The authors should acknowledge this limitation more explicitly and suggest potential stronger gold-standard validity benchmarks.
13. The data availability statement indicates datasets are available upon request. This does not fully align with PLOS ONE’s data policy, which generally requires deposition in an open repository unless there are compelling reasons. Authors should ensure raw scoring data, item-level assessments, and country-level evaluations are made available.
14. The conclusion overstates the robustness of the instrument. Given some domains had low reliability and the pilot sample was limited, claims about wide applicability should be more cautious. The authors should emphasize that further validation in diverse contexts is necessary. Revise the conclusion accordingly.
15. Separately add the limitations and future recommendations section below the conclusion part, clearly mentioning the drawbacks of the current study, and what next can be done, discus this in a logical way.
16. The manuscript is generally clear, but some sections (Methods, particularly instrument development and response scale generation) are overly technical and could be streamlined for readability. A flowchart summarizing instrument development steps would help.
17. Table 3 (response scales) is long and dense; it may be better placed in supplementary materials. Figures 1–3 could use clearer captions that describe the key interpretation points (e.g., what higher/lower scores mean in context).
18. The manuscript uses “AFR” and “Accountability for Reasonableness” interchangeably. Standardize terminology to avoid confusion.
19. Several references are dated (e.g., 2001, 2002 WHO guidelines). More recent WHO documents (2023 model list and guidance) should be cited where applicable.
20. Appendix A, Pilot Study Sample Countries: Only four countries are included, which limits representativeness, the table does not explain why these specific countries (e.g., Afghanistan vs. other low-income countries) were chosen. Explicitly state the selection rationale beyond income-stratification (e.g., data availability, policy diversity). My recommendation is that discuss these points as a footnote or provide a brief explanation in discussion part.
21. Appendix B, Conversion Calculation: No worked example is provided, making it difficult for readers to follow how conversion works in practice. What was the logic behind choosing *42 as the total raw max score* and converting to *80* is not sufficiently explained. I recommend to Include a step-by-step example for one item and country, showing raw score.
22. Appendix C, Explanations are superficial
23. Appendix D, Clarify the meaning of rater codes, add a **legend**, and consider averaging scores in a separate summary table.
24. Appendix E, Adds little beyond what is already in Results.Unclear whether these are averages across raters or consensus scores. State clearly if the totals are mean scores. If redundant with main text, move to supplementary-only.
25. Appendix F, Only percentages are provided; no statistical measures (correlation coefficients, regression outputs) are included. Proxy measures mainly capture *timeliness* of updates, not full process quality. Include statistical tests (e.g., Pearson/Spearman r, regression slopes). Acknowledge limitations of proxies more strongly.
26. Appendix G, Some domains show poor reliability (e.g., Enforcement ICC = 0.44, CI includes negative values). This is downplayed in the main text. The appendix does not explain why enforcement reliability is so weak (likely due to single-item measure). Add an explicit note on interpretability issues for domains with poor reliability. Consider revising the instrument to include more items under weak domains.
27. The appendices add value by making the methodology transparent, but they currently fall short of journal-quality supplementary material. They require: Better formatting (clearer tables, legends, consistent decimals), Stronger statistical reporting (validity beyond descriptive percentages). More detailed justification for methodological decisions (item reduction, country selection). Removal of duplicated or redundant sections. Without these revisions, the appendices risk confusing rather than supporting readers.
28. Major Revision Required: The manuscript is promising and fills an important methodological gap, but revisions are required to strengthen justification of the study design, address limitations of reliability/validity, clarify ethics and data availability, and moderate conclusions.
